# Conventional versus video-assisted laryngoscopy for perioperative endotracheal intubation (COVALENT) - a randomized, controlled multicenter trial

**DOI:** 10.1186/s12871-023-02083-3

**Published:** 2023-04-18

**Authors:** Benedikt Schmid, Dominik Eckert, Andreas Meixner, Paul Pistner, Uwe Malzahn, Monika Berberich, Oliver Happel, Patrick Meybohm, Peter Kranke

**Affiliations:** 1 Department of Anaesthesiology, Intensive Care, Emergency and Pain Medicine, University Hospital Würzburg, Oberdürrbacher Str. 6, 97080 Würzburg, Germany; 2 Clinical Trial Center, University Hospital Würzburg, Josef-Schneider-Str. 2, 97080 Würzburg, Germany

**Keywords:** Anaesthesiology, Laryngoscopy, Video-assisted laryngoscopy, Intubation, Airway management, Patient safety, Human factors

## Abstract

**Background:**

Data on the routine use of video-assisted laryngoscopy in peri-operative intubations are rather inconsistent and ambiguous, in part due to small populations and non-uniform outcome measures in past trials. Failed or prolonged intubation procedures are a reason for relevant morbidity and mortality. This study aims to determine whether video-assisted laryngoscopy (with both Macintosh-shaped and hyperangulated blades) is at least equal to the standard method of direct laryngoscopy with respect to the first-pass success rate. Furthermore, validated tools from the field of human factors will be applied to examine within-team communication and task load during this critical medical procedure.

**Methods:**

In this randomized, controlled, three-armed parallel group design, multi-centre trial, a total of more than 2500 adult patients scheduled for perioperative endotracheal intubation will be randomized. In equally large arms, video-assisted laryngoscopy with a Macintosh-shaped or a hyperangulated blade will be compared to the standard of care (direct laryngoscopy with Macintosh blade). In a pre-defined hierarchical analysis, we will test the primary outcome for non-inferiority first. If this goal should be met, the design and projected statistical power also allow for subsequent testing for superiority of one of the interventions.

Various secondary outcomes will account for patient safety considerations as well as human factors interactions within the provider team and will allow for further exploratory data analysis and hypothesis generation.

**Discussion:**

This randomized controlled trial will provide a solid base of data in a field where reliable evidence is of major clinical importance. With thousands of endotracheal intubations performed every day in operating rooms around the world, every bit of performance improvement translates into increased patient safety and comfort and may eventually prevent significant burden of disease. Therefore, we feel confident that a large trial has the potential to considerably benefit patients and anaesthetists alike.

**Trial registration:**

ClincalTrials.gov NCT05228288.

**Protocol version:**

1.1, November 15, 2021.

## Background

### Rationale

Endotracheal intubation is the gold standard for airway management under general anaesthesia. Over the course of the past several years, video-assisted laryngoscopy (VAL) has more and more gained in importance in this field and has already found its way into the relevant guidelines for certain settings and situations like the unexpected difficult airway [1], critically ill patients [2] or prehospital airway management [3]. Although VAL is attested superiority especially in unexpected difficult airways, known difficult airways, and obese patients in some studies [4–6], inconsistent results in the different studies and sometimes very small patient populations make clear recommendations difficult apropos further generalisability [5].

While video laryngoscopes already significantly expand the field of view simply by positioning the camera, hyper-angulated blades can also accommodate significantly greater curvatures of the pharyngeal anatomy of certain patients where a direct view of the glottis plane would otherwise not be possible [7]. This has further increased success rates in selected patient collectives.

VAL has tended to be superior to direct laryngoscopy in terms of safety in the studies conducted to date, but the quality of evidence based on the existing studies is still insufficient to draw definitive conclusions [5]. Thus, despite the enormous clinical relevance of the question [8, 9], insufficient solid data currently exist to make conclusive recommendations for or against the standard use of VAL in daily anaesthesiologic practice. One reason for the insufficient evidence certainly is the frequently inconsistent or inappropriate selection of endpoints in previous studies [10–13].

A Cochrane systematic review with a total of 64 included studies concluded that primary endotracheal intubation using a video laryngoscope leads to a lower number of failed attempts in emergency scenarios, especially in patients with an expectedly difficult airway [5]. However, the evidence listed for this finding according to the Grading of Recommendations, Assessment, Development and Evaluations (GRADE) system is reported to be only moderate. Furthermore, it is indicated that VAL can be used to improve the view of the glottis and vocal fold plane. VAL could achieve significantly lower Cormack & Lehane scores [14] and reduce the number of grades 3 and 4, equivalent to significantly poorer glottic visibility. A significant improvement of the Cormack & Lehane grade using VAL was also confirmed in a multi-centre study by Kaplan et al. [15]. In addition, VAL has been attributed with potential advantages in terms of handling and a lower incidence of mucosal, laryngeal, and tracheal damage, as well as a lower incidence of hoarseness compared to conventional laryngoscopy [16]. In contrast, no statistically significant differences are described between the different devices with respect to either the first-attempt success rate or the total number of intubation attempts required. The same applies to the occurrence of hypoxia and other respiratory complications. The authors point out that the evidence for these findings must be considered very low due to a lack of robust data. Moreover, many endpoints, such as time to successful intubation, are difficult to evaluate in a meta-analysis due to heterogeneous definitions and lack of comparability.

VAL has been included in the guidelines for airway management of the respective professional societies with different recommendations. For the perioperative setting, VAL is mentioned as a back-up procedure in the German guidelines, e.g. in case of an unexpectedly difficult airway after max. two conventional laryngoscopy attempts as well as in obese patients [13]. In the field of obstetric anaesthesia, the recent S1 guideline calls for VAL to be available and also considers its use as a primary device to be an option: “A video laryngoscope can facilitate airway management in pregnant women in the context of an RSI and may also be considered as a first-choice method” [17]. In contrast, the current guideline on prehospital emergency anaesthesia with endotracheal intubation recommends the use of a video laryngoscope as the primary tool [3]. In current meta-analyses, primary success rates for endotracheal intubation are regularly reported to be slightly higher for VAL (> 90%) than for conventional laryngoscopy (< 90%) [4, 6, 16, 18].

Going beyond the mere procedure of endotracheal intubation, i.e. inserting a tube in the trachea, this study also aims to descriptively investigate the domain of human factors in the context of critical medical interventions using the example of airway management. In general, this aspect, which examines psychological and physiological influences (among others) on process functioning, is still largely left out of anaesthesiologic research. However, a 2013 study concluded that in all cases in which anaesthesiologists had reported serious airway rescue incidents to a relevant registry, such human factors had contributed to the errors that occurred [19]. In the context of intensive care medicine [20, 21] or simulation training [22], individual reports have been published so far, but data from large collectives are still lacking. For this reason, in this study, after each intervention, the performing anaesthesiologist and the collaborating nurse will be asked to complete two brief, validated questionnaires to capture human factors-relevant observations and assessments: The Mayo High Performance Teamwork Scale (MHPTS) [23] and the NASA Task Load Index (NASA-TLX) [24].

The MHPTS consists of 16 individual items that describe a representative number of characteristic nontechnical crew resource management (CRM) behaviours for high-performance teamwork and can be rated using a 3-point scale (0 = never/almost never, 1 = alternating/inconsistent, 2 = constant/consistent). It thus provides a brief, validated, and practical rating scale that can be used by CRM-inexperienced team members in a high-performance medical setting to reflect on important CRM skills and evaluate their own team performance. The NASA-TLX assesses subjectively perceived stress while completing a task on a visual analogue scale (VAS) in six domains [24]. The NASA-TLX is widely used in the field of human factors research and has been used in scientific studies in the field of surgery, for example [25].

### Hypothesis and experimental approach

Based on the data available to date, it can be assumed that VAL is at least equivalent to direct laryngoscopy also in the context of elective intubations but could increase patient safety in special situations. Therefore, this trial implements a three-arm, parallel group, randomized-controlled design to investigate the hypothesis that VAL is primarily non-inferior to conventional laryngoscopy in terms of the rate of successful intubations at first attempt. In this study, patients are randomized to one of three parallel study arms: in the first arm, the patient is primarily intubated using conventional, direct laryngoscopy with a Macintosh blade. This intervention, thus far considered the gold standard, also represents the control group. In a second arm, patients will be intubated by VAL, also using a Macintosh-shaped blade. In the third study arm, the primary instrument to be used will be a video-assisted laryngoscope, where primarily and regardless of possibly known difficult airway, a hyper-angulated blade will be used.

We hypothesize that the procedure “video-assisted laryngoscopy”, i.e. both VAL study arms taken together in a first analysis step, is non-inferior to the “conventional laryngoscopy” control group regarding the primary endpoint “successful intubation at first attempt”. If this first attempt fails, the decision on the laryngoscopy procedure to be used in the further course is the sole responsibility of the anaesthesiologist performing the procedure; for reasons of patient safety, the study protocol does not contain any provisions in this regard, and the procedure primarily used according to randomization can be abandoned without consequence for study participation.

### Benefit-risk assessment

All patients receive the medically necessary peri-operative care. According to current knowledge, randomization into one of the three study arms does not per se entail any altered risk for participants. Both conventional laryngoscopy and VAL are established as standard clinical procedures. The fact that, according to the current state of knowledge, no procedure is superior to the other for elective endotracheal intubation in the context of general anaesthesia is precisely the subject of this study. There is no evidence from the data available in the literature to suggest that VAL may expose the patient to additional risk compared with the conventional procedure.

Since both investigated laryngoscopy procedures are used in routine clinical practice, but sufficient robust data to demonstrate the superiority of either procedure have been lacking to date, the investigation of this issue in a randomized-controlled clinical trial with the aim of maximizing patient safety during a potentially life-threatening intervention also appears to be ethically appropriate. No increased risk results for the patient from participation in this clinical study, neither is a direct medical benefit for the patient assumed resulting from participating.

### Choice of comparators

**Fig. 1:**
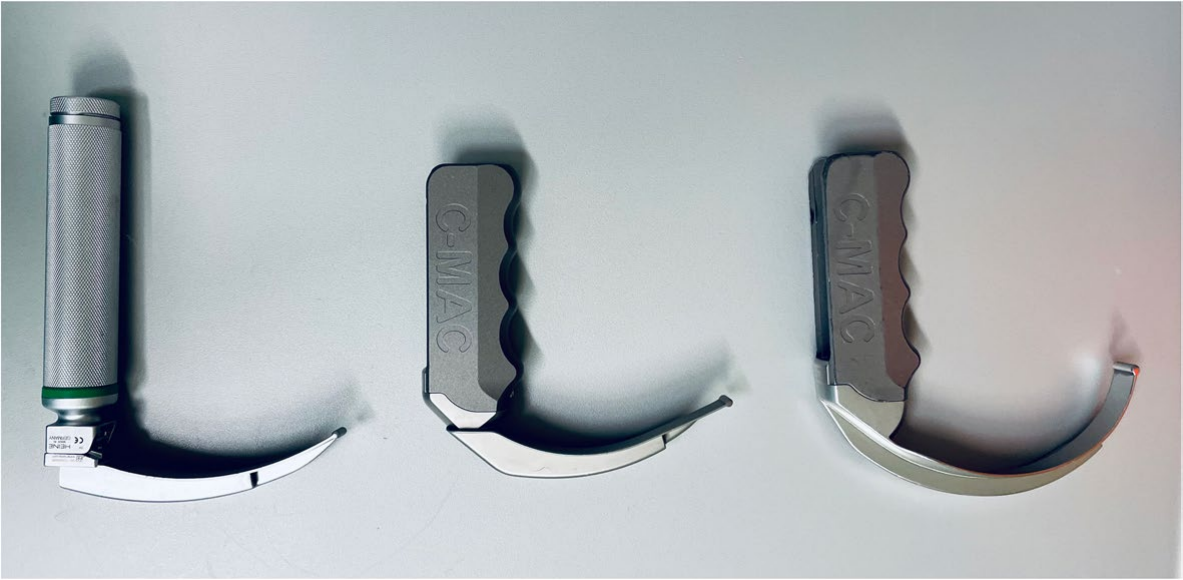
Examples of different laryngoscopes to be used in each of the three study arms (from left to right): conventional laryngoscope with Macintosh blade, video-assisted laryngoscope with Macintosh-shaped blade, video-assisted laryngoscope with hyper-angulated blade

In this study, three different modalities of laryngoscopy for endotracheal intubation will be compared (s. Fig. 1): 1. conventional, direct laryngoscopy using a Macintosh blade. This procedure is considered the gold standard and is routinely used in the peri-operative setting as well as in intensive care and emergency medicine. 2. use of VAL with a Macintosh-shaped blade. By positioning the camera at the tip of the blade, the field of view onto the glottic plane is improved, as there is no need to establish a direct line of sight from the outside to the tracheal inlet (as is the case with conventional laryngoscopy). 3. use of VAL with a hyper-angulated blade. This shape of laryngoscope blade cannot be used in conventional, direct laryngoscopy because the strong curvature renders a direct line of view from the outside impossible due to the geometry of the instrument. So far, hyper-angulated blades are often used as a backup instrument when a view of the glottic plane cannot be achieved with the Macintosh blade due to anatomic abnormalities, even with a video-assisted laryngoscope. This often enables endotracheal intubation in patients who could otherwise not be intubated through conventional laryngoscopy. At the same time, however, it is conceivable that the rather extreme curvature of the blade may prove disadvantageous in individual cases compared with less curved blade variants. This may be because insertion of the tube is difficult due to the curvature of the blade and indirect vision, or because fluids like mucus or blood make it impossible to see through the video-optical system.

After testing the non-inferiority of the experimental intervention (VAL as an overall procedure) compared with the control group, pairwise comparisons between the study arms will also be of interest. If this step of the hierarchical analysis plan can be reached, testing for superiority between groups will be possible. Furthermore, the question of whether the primary use of a hyper-angulated blade may be useful can be explored. In this scenario, for example, the hyper-angulated blade would not be inferior in the general setting, but would possibly make a second intubation attempt, or a change of the device used, unnecessary in the case of an unexpectedly difficult airway, since the “rescue device” has already been used to begin with.

### Aims of the study

#### Primary aim

The primary aim of this trial is to study whether VAL is non-inferior to conventional laryngoscopy for endotracheal intubation in the context of elective surgery under general anaesthesia. Non-inferiority hereby refers to the average rate of successful intubations at first attempt from all interventions in a study arm.

#### Further aims

In addition, several other parameters will be collected, which, individually or in combination, can provide insights into the risk–benefit profile of the interventions examined. In case of previously proven non-inferiority as laid out in the primary hypothesis, substantial differences between the procedures regarding accompanying injuries, critical changes in vital parameters during intubation, etc., may hint towards a general advantage of one procedure over the others.

Furthermore, perceived task load and the quality of the information flow within the team of care providers (mostly anaesthetists and specialist nurses) shall be assessed using structured, validated questionnaires. The underlying hypothesis is that VAL, through the progress of the intubation process being visible to all involved, simplifies supportive or anticipatory measures by the specialist nursing staff and improves the non-verbal information flow.

Ultimately, the primary intention of this study is to determine whether there is a generally recommended instrument that is non-inferior in the uncomplicated airway but possibly causes fewer complications in the (unexpected) difficult airway.

## Methods and design

### Design

**Fig. 2:**
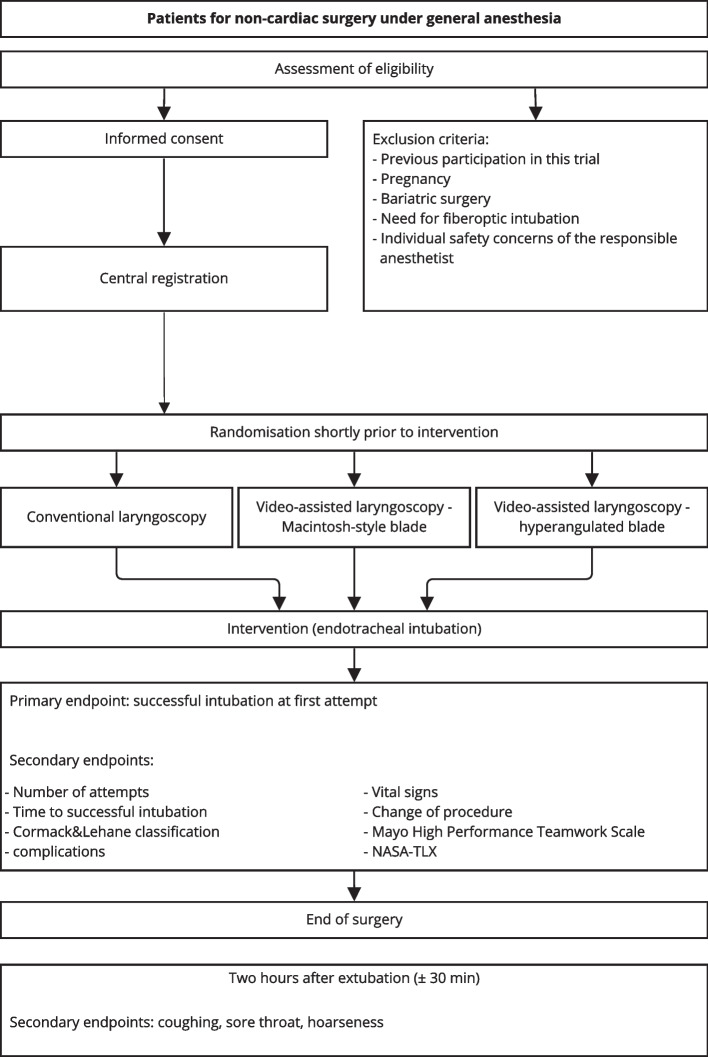
Flow diagram

This is a randomized controlled multi-centre study with a three-arm parallel study design (s. Fig. 2). The primary endpoint is the successful placement of the endo- tracheal tube at first attempt (success at first attempt) as a dichotomous feature. The patients included in the study will be evenly distributed across the three study arms (1:1:1). The underlying approach is the premise of non-inferiority of the experimental interventions compared to the established standard procedure.

The “conventional laryngoscopy” study arm serves as a control group in this experimental setup. The two study arms using VAL will initially be combined at the first stage of the analysis to show that the method “VAL” is non-inferior to the established gold standard regardless of the blades used. If this first analysis can prove non-inferiority, the three individual groups will also be compared against each other.

## Methods

### Setting

The main trial site is the Department of Anaesthesiology, Intensive Care Medicine, Emergency Therapy and Pain Therapy at the University Hospital of Wuerzburg. Further participating sites will be located at the departments of anaesthesiology of hospitals of primary and maximum care in Germany, Austria and Switzerland. Data collection will take place during the induction of anaesthesia prior to elective surgical procedures under general anaesthesia and, after the operation, in the peri-anaesthesiologic ward.

Inclusion criteria.

- Adult patients
- Scheduled for elective, non-cardiac surgical procedure
- Need for endotracheal intubation as determined during the premedication visit

Care providers performing the intubation will have at least one year of training in anaesthesiology and sufficient experience in the use of all devices tested.

Exclusion criteria.

- Previous participation in this study
- Pregnancy
- Need for fibreoptic intubation
- Patients scheduled for bariatric surgery
- Any circumstance that will lead the anaesthesiologist(s) in charge to believe (before randomization)

That random assignment of a laryngoscopy instrument may compromise patient safety during induction of anaesthesia.

### Interventions

The preparations for induction of anaesthesia as well as establishing the necessary patient monitoring will take place in accordance with the stipulations of the anaesthetist in charge and with local standards. Randomization will take place electronically on the day of the intervention; the randomization result is communicated to the team performing the intervention (i.e. anaesthetist and anaesthesia nurse) in such a way that the patient is unaware of it. The study protocol does not specify the manufacturer or model of the laryngoscope to be used (e.g., disposable vs. reusable blades). However, the kind of blade will be recorded, as previous research suggested considerably reduced glottic view with (disposable) plastic blades compared with metal blades [26].

The choice and dosage of the anaesthetics as well as the sequence of administration is up to the decision of the anaesthetist in charge. For better comparability between the interventions, adequate pre-oxygenation (etO2 > 80%) and relaxation of the patient (ideally, train of four (TOF) = 0) is required before starting the laryngoscopy. The process of laryngoscopy (and thus also the first attempt at intubation) begins when the blade of the laryngoscope passes the patient’s row of teeth for the first time. The trial observer will also start the time measurement for the intubation procedure at this timepoint. The intubation is considered to have been completed successfully when the first capnographic end-expiratory CO2 detection is possible after the endotracheal tube has been placed. In the event of failure, the first attempt at intubation is deemed to have ended at the point in time when either the laryngoscope (e.g., after inadequate visibility of the vocal cords) or the endotracheal tube is removed from the patient’s mouth. Each additional intubation attempt using the same criteria should be considered a completed attempt.

The use of an intubation stylet is not primarily intended; the exception here is the use of a hyper-angulated blade, which cannot be used properly without a stylet. The routine use of a stylet for every endotracheal intubation is discussed in the literature in the context of critically ill patients [27], but ultimately does not reflect common practice in elective, peri-operative intubations. The use of a stylet after the start of the first intubation attempt with the other two types of blades is considered a change of device and thus also a failure of the first intubation attempt. If the anaesthetist in charge using a (video) laryngoscope with a Macintosh blade chooses to use a stylet for whatever reason before the start of the first attempt at intubation, this will be tolerated as per the study protocol.

After completion of the intervention, both the nurse and the anaesthetist in charge will answer the MHPTS questionnaire and the NASA-TLX questionnaire. To ensure that no necessary medical measures are delayed or hindered under any circumstances will be of the essence.

### Outcomes

The primary endpoint is the rate of successful intubations at first attempt as a dichotomous (successful/unsuccessful) event. The first attempt starts with the laryngoscope blade passing the patient’s row of teeth. The attempt is considered unsuccessful if a complete retraction of the laryngoscope or the endotracheal tube from the oropharynx is necessary for any reason (need for bag ventilation, change of device, change of patient position, change in the curvature of the stylet, etc.). Manoeuvres that can be performed during laryngoscopy, such as BURP or reclining the head, which end with successful intubation, count towards the first attempt.

Secondary outcomes are as follows:

1. Parameters regarding the duration of the intervention
  time to successful intubation (i.e. from start of first intubation attempt to first positive capnography via an endotracheal tube).
  time to first glottic view (as announced by the anaesthetist)
2. Cormack & Lehane grade
3. Intubation success:
  total number of attempts
  number of attempts using the randomized device
  success rate of intubation attempts regarding the respective device
4. Influence of the device on human factors during the intervention
  Mayo High Performance Teamwork Scale
  NASA-TLX
5. Complications: occurrence of one or more of the following
  Drop of SpO2 below 90%
  Regurgitation as announced by the anaesthetist
  Need for bronchoscopy due to suspected aspiration
  Dental injury or dental clicks upon contact with the blade
  Soft tissue injuries as detected by blood on the blade
  Visible swelling, bleeding or other injury of the lips
  Need for resuscitation (administration of adrenalin, chest compressions, defibrillation or any combination thereof)
  Death
  Occurrence of any adverse event (AE) or serious adverse event (SAE)
6. Need for auxiliary devices or switch of laryngoscopy device
  Manoeuvres to optimize intubation conditions after the first attempt to intubate the patient has started
  Switch to alternate laryngoscopy device
  Switch of the anaesthetists
  Switch of airway device
7. Relevant vital parameters during the intervention: baseline SpO2 and lowest value during the airway procedure
8. Post-operative sore throat, coughing or hoarseness: each rated from 0 to 3, score adapted from Park et al. [27]. This outcome will not be assessed in patients who continue to be invasively ventilated >2 hours after surgery.

Further patient-related parameters to be collected are:

- Age
- Gender
- Height and weight
- ASA classification
- Mallampati score
- upper lip bite test (ULBT) classification

Where applicable, these can be obtained from the patient’s chart.

Care provider-related data to be collected are:

- Job experience (years; anaesthetist only)

The observable outcomes will in part be captured using the Work Observation Method By Activity Timing (WOMBAT) software (WOMBAT 3.0, 2020) [28].

Schedule of activities.

See Table. 1

**Table 1:** Schedule of activities

	Screening	randomization	intervention	post-intervention	follow-up
	up to 14 days prior to intervention	day 0 briefly before intervention	day 0	day 0 immediately after intervention	day 0 2 h (± 30 min) after extubation
visit	V1	V2	V3	V4	V5
inclusion criteria	X				
exclusion criteria	X				
informed consent	X				
registration	X				
sociodemographics, medical history	X				
acute medical concerns by responsible anaesthetist preventing randomization		X			
randomization		X			
experience of *anaesthetist*			X		
upper lip bite test, Mallampati score	X		(X)^a^		
pre-oxygenation (> 80% etO_2_), relaxation (TOF = 0)			X		
intubation (incl. number of attempts and time to intubation)			X		
Cormack-Lehane grade			X		
switch of device or provider			X		
vital signs			X		
injuries			X		
Mayo High Performance Teamwork scale				X	
NASA-TLX				X	
sore throat, coughing, hoarseness					X

^a^ if these parameters cannot be obtained from the patient’s chart, they will be obtained by the observer prior to the intervention

### Sample size

The estimation of the appropriate sample size is based on the corresponding Null hypotheses of the primary hypothesis, i.e. the assumption that VAL is inferior to conventional laryngoscopy in terms of first-attempt success rate, irrespective of the kind of blade in use. From clinical experience and previously published data, we assume an a-priori success rate of 90% across all groups. A clinically relevant difference between groups in this context is suggested at 5%, as is the accepted overall type-I error. Given that both Null hypotheses will be tested separately, the type-I error limit in each case is 2.5%. The desired statistical power is set to 90%. The Null hypotheses will be tested for non-inferiority using a one-sided Z test with unpooled variance. Based on these assumptions, the required sample size amounts to 3 * 824 = 2526 participants.

### Recruitment

Given the large number of surgical interventions performed under general anaesthesia at the study centres involved each day and only little restrictive inclusion and exclusion criteria, insufficient numbers of potential study participants are not to be expected. Because the additional burden on the participants while participating in the study is deemed to be manageable, a high recruitment rate can be assumed. We do not expect substantial problems with protocol adherence due to the short duration of the intervention.

Randomization takes place automatically through computer-aided generation of random numbers, which are translated into a randomization decision and will be triggered online by the respective investigator on the day of the intervention at the earliest, so that a concealed allocation to the interventions is ensured (allocation concealment). The randomization result will be communicated non-verbally to the anaesthetist and the nursing staff involved via a display on the tablet monitor to ensure that the patient remains blinded.

The generation of the allocation sequences and the allocation to the individual intervention arms takes place via an IT-based system (OpenClinica, Waltham, MA, USA) coordinated by the Center for Clinical Studies of the German Association of Anaesthesiology and Intensive Care (DGAI).

If the anaesthetist in charge of the intervention decides not to leave the choice of the intubation instrument to chance for medical reasons before the induction of anaesthesia and prior to randomization, the participation in the trial for this patient and the case will be considered a “screening failure”. If such a decision (deviating from the randomization result) is made after the patient has been randomized but before the start of the first intubation attempt, the first intubation attempt will be.considered to have failed. These cases will be reported separately in the study flow chart. The remainder of intervention as per protocol remains unaffected. After a first intubation attempt with the randomized laryngoscopy device, the anaesthetist performing the procedure is free to change the instrument at his/her own discretion. The required intubation attempts and the time to successful intubation are recorded as described above.

In addition, the study protocol does not make any specifications for handling possible complications in securing the airway. The physician carrying out the intervention always has unrestricted authority to make medical decisions to prevent harm to the patient without affecting the patient’s participation in the study.

### Blinding

Patients and data analysts will be blinded regarding the allocation of interventions. The randomization result will be communicated to the care providers non-verbally in such a way that the patient will not take note. Before data will be evaluated, the identifying variable names will be made unrecognizable. For the study observers and the personnel carrying out the intervention, blinding is not possible due to the nature of the study. In the follow-up period two hours after the end of anaesthesia, the survey of the outcomes should be carried out, if possible, by an observer who was not involved in the intervention, to encourage blinding of the investigator here as well. It will be documented whether blinding could be facilitated at this point.

Un-blinding of the patient is intended only in cases when an unexpected difficult airway situation occurred, and the patient needs to have an anaesthesia problem card issued for them. However, even in such cases, blinding can usually be kept up until after the last study visit.

### Biometry

The required socio-demographic data as well as data on the surgical procedure will be assessed by chart review beforehand and will be included in the study documentation as source data. All other necessary data are determined and recorded by the investigator or observer during the intervention in real time. The questionnaires on teamwork and task load will be filled out by the care providers as soon as the ongoing procedures allow for. The principal investigator at each study site will be responsible for the timely documentation in the electronic case report form (eCRF) and the proper documentation and storage of source data according to applicable local laws.

All data relevant to the trial will eventually be entered into an eCRF and stored in an electronic database provided by OpenClinica LLC (Waltham, MA, USA). Hosting of the database in accordance with German and EU laws will be ensured by Docs In Clouds Telecare GmbH (Aachen, Germany).

Data collection forms can be requested from the authors.

### Statistical analysis

The primary outcome is the rate of successful intubations at first attempt as a fraction of dichotomous events. In a first analysis step we test the hypothesis, that neither video assisted laryngoscopy with a Macintosh-shaped blade (M-VAL) nor video assisted laryngoscopy with a hyper-angulated blade (H-VAL) are inferior to conventional direct laryngoscopy (CDL) with regard to the primary outcome. In terms of the statistics, this corresponds to testing the Null hypothesis that at least one of the interventions M-VAL or H-VAL is inferior to CDL. Both Null hypotheses will be tested for separately. The overall type-I error will be set to 5%. Thus, both Null hypotheses will be tested on a local significance level of 0.025 using a one-sided Z-test with unpooled variance. Non-inferiority is defined as described in subsection “sample size”.

Only if both Null hypotheses can be rejected will there be a second analysis step where the single interventions M-VAL and H-VAL will be tested pairwise for superiority using a two-sided Z-test with unpooled variance. As we apply the principle of fixed sequence hierarchical testing, this analysis can be performed at a local significance level of 0.05 to meet the family-wise error rate of 0.05.

A planned interim analysis will be conducted after 200 randomized interventions. This will first and foremost happen to review feasibility and data quality before expanding to additional study sites. We do not foresee any substantial safety concerns in this trial which would need to be assessed at this stage.

### (Serious) Adverse events (SAE / AE)

As all kinds of laryngoscopes and blades used in this study are also widely used in routine clinical practice, we do not expect any trial-specific AEs. However, any definitive failure to successfully place an endotracheal tube using the allocated or subsequently any other device deemed suitable shall be documented as an AE. This could be the case when a supraglottic airway device is used without further attempts to facilitate endotracheal intubation or when anaesthesia is being terminated early while bag-and-mask-ventilating the patient.

An SAE is present if one or more of the following occur:

- Death during the observation period
- Vital threat to the patient
- Need for previously not planned in-patient treatment or prolongation thereof
- Occurrence of persisting or severe disability

AEs and SAEs must be reported to the sponsor as soon as possible and be recorded in the eCRF.

### Ethics, data privacy and data availability

All activities within this trial comply with the Declaration of Helsinki and the principles of Good Clinical Practice (GCP).

### Ethics approval

The German version of this protocol was approved by the Ethics committee at the Medical Faculty, University of Wuerzburg, Germany (No. 215/21-sc). Any protocol amendments or supplements require review by the responsible ethics committee and will be communicated to the study centres after approval by the committee.

### Informed consent

Informed consent will be obtained by trained investigators at the respective study sites. The necessary information material and consent forms will be provided by the sponsor of the trial. Patients who are not capable of giving consent and those under legal custody are excluded from participation in the study.

### Data privacy

Personal data of patients are collected only to the extent required by the study protocol. All data collected through observation of the intervention will be collected in a pseudonymized manner. Only excerpts from the anaesthesia protocol for documentation of vital signs during the intervention will be directly assignable to individual patients and will be processed and stored in accordance with the applicable national and EU data protection regulations and the principles of Good Clinical Practice. Data of the care providers will be collected anonymously. Detailed information for participants on collecting, processing, and storing trial data will be part of the patients’ information brochure.

### Data availability

The final data set will remain property of the sponsor. Other parties will not be entitled to have access to the data. The sponsor reserves the right to make individual or all primary data available in anonymized form to other scientists upon request for the purposes of reviewing the conducted and performing own subsequent analyses.

Publication of the results of this study in peer-reviewed medical journals will be pursued. Authorship guidelines as put forth by the International Committee of Medical Journal Editors (ICMJE) will be adhered to.

### Roles and responsibilities

In this investigator-initiated trial, the trial sponsor is involved in the study design. Researchers concerned with the preparation of this protocol, data collection and eventually seeking to publish the results are in part employed by the sponsor. The sponsor retains ultimate authority over these activities.

## Discussion

We propose here the protocol for a randomized, controlled, patient-blinded multi- centre trial on the routine use of VAL in a peri-operative anaesthesiologic setting. Although several studies in the broader research area have been conducted in the past, conclusive evidence enabling clear recommendations is still not available. As Lewis et al. pointed out in a Cochrane systematic review on the matter, there is high heterogeneity in the findings [5]. However, all studies included in this systematic review have included rather small cohorts given the context of the research question. For example, the trial with the most patients per study arm assessed 264 patients in the intervention group and 256 in the control group [29]. Recently, *Kriege *et al*.* published preliminary data in abstract form from a multicentre RCT including more than 2100 patients [30]. Therein, VAL was superior to direct laryngoscopy in terms of first pass success. However, the trial was limited to one manufacturer of video laryngoscopes and did not consider hyper-angulated blades. Full data or detailed analyses have not been presented yet. Considering the sample size calculation in our protocol, one must assume that to date, there has not been a sufficiently powered trial addressing the question at hand.

Despite our best efforts to design this study as diligently and efficiently as possible, some points of concern still remain. For one, there will always be an issue with the inability to blind the interventionist regarding the intervention allocation. It is well conceivable that the physician performing endotracheal intubation will always be aware of the device they are using. In analogy to this, the outcome assessor cannot be blinded for similar reasons. Therefore, there will always be a risk for performance or detection bias. We will, however, have patients and statisticians blinded, thus avoiding additional potential bias where possible.

With a recruitment goal of more than 2,500 patients, we needed to assure that the observation period stays as compact and short as possible. To meet a comprehensive set of outcome parameters which allows for proper comparability with previous studies, we incorporated in this trial a follow-up two hours after the end of surgery to assess additional outcomes like hoarseness and sore throat. However, we decided not to extend the follow-up period for possible endpoints like hospital length of stay or mortality. We do not foresee any fundamental issues or inconveniences in recruiting the projected number of patients. Due to the nature of the inclusion and exclusion criteria, many patients will be eligible for participation and with a sufficiently large number of additional study sites we expect to meet the recruitment goal within two years. This is needed to ensure that the accompanying circumstances remain reasonably constant during the conduct of the study.

With the results from this trial, we aim to contribute a substantial foundation of evidence for the use of VAL in the routine peri-operative setting. We intentionally designed this study as a non-inferiority trial. In a mostly unselected population like this one, overall first attempt success rates for intubation are (fortunately) high to begin with. We cannot with reasonable certainty expect to see VAL superior in this regard, although with the choden statistical design this would be possible to prove as well. However, with the ORs of patient-relevant secondary outcomes like laryngeal/airway trauma, sore throat and hoarseness previously reported to tend to favour VAL (OR [95% CI] 0.68 [0.48, 0.96], 0.82[0.56, 1.19] and 0.57 [0.36, 0.88], respectively [5]), even if statistically non-inferior VAL may be beneficial for patients in this overall constellation. Furthermore, we see the necessity to promote the awareness of human factors in high-risk procedures like airway management. Understanding how team dynamics change depending on the design of the devices they are using may help to promote the idea of ergonomic engineering in the medical field. Hence, we believe this will provide a valuable basis for the generation of future hypotheses.

### Funding

This trial is funded through departmental funds of the Department of Anaesthesiology, Intensive Care, Emergency and Pain Medicine Pain, University Hospital Wuerzburg, and supported by the German Society for Anaesthesiology and Intensive Care.

## Data Availability

Data will be available upon request from the corresponding author after completion of the trial. A model consent form and patients’ information as well as the Mayo High Performance Teamwork Scale and NASA-TLX questionnaires (all in German) as used in this trial can be obtained from the authors upon request.

## Abbreviations

AE: Adverse event
ASA: American Society of Anesthesiologists
BURP: Backward, upward, rightward pressure
CDL: Conventional direct laryngoscopy
CI: Confidence interval
CRM: Crew resource management
eCRF: Electronic case report form
GCP: Good clinical practice
GRADE: Grading of recommendations, assessment, development, and evaluations
H-VAL: Video-assisted laryngoscopy with hyperangulated blade
ICMJE: International Committee of Medical Journal Editors
M-VAL: Video-assisted laryngoscopy with a Macintosh-shaped blade
NASA-TLX: NASA task load index
OR: odds ratio
RSI: Rapid sequence induction
SAE: Serious adverse event
TOF: Train of four
ULBT: Upper lip bite test
VAL: Video-assisted laryngoscopy
VAS: Visual analogue scale
WHO: World Health Organization
